# Cancer Immunotherapy Using γδT Cells: Dealing with Diversity

**DOI:** 10.3389/fimmu.2014.00601

**Published:** 2014-11-20

**Authors:** Wouter Scheper, Zsolt Sebestyen, Jürgen Kuball

**Affiliations:** ^1^Laboratory of Translational Immunology, Department of Hematology, University Medical Center Utrecht, Utrecht, Netherlands

**Keywords:** γδT cells, cancer immunotherapy, γδT cell diversity, innate-like lymphocytes, γδTCR

## Abstract

The broad and potent tumor-reactivity of innate-like γδT cells makes them valuable additions to current cancer immunotherapeutic concepts based on adaptive immunity, such as monoclonal antibodies and αβT cells. However, clinical success using γδT cells to treat cancer has so far fallen short. Efforts of recent years have revealed a striking diversity in γδT cell functions and immunobiology, putting these cells forward as true “swiss army knives” of immunity. At the same time, however, this heterogeneity poses new challenges to the design of γδT cell-based therapeutic concepts and could explain their rather limited clinical efficacy in cancer patients. This review outlines the recent new insights into the different levels of γδT cell diversity, including the myriad of γδT cell-mediated immune functions, the diversity of specificities and affinities within the γδT cell repertoire, and the multitude of complex molecular requirements for γδT cell activation. A careful consideration of the diversity of antibodies and αβT cells has delivered great progress to their clinical success; addressing also the extraordinary diversity in γδT cells will therefore hold the key to more effective immunotherapeutic strategies with γδT cells as additional and valuable tools to battle cancer.

## Immunotherapy to Treat Cancer: The Era is Now

Current treatment options to fight cancer heavily rely on pharmaceutical and radiological interventions that are accompanied by substantial off-tumor toxicity and lack of clinical efficacy. Cancer immunotherapy aims to capture the specificity and memory of the immune system and holds the promise of truly targeted treatment with durable clinical responses. Recent advances in clinical trials and the approval of more and more immunotherapeutic agents by international regulatory agencies have given the field considerable momentum, a fact that is mirrored by the announcement of cancer immunotherapy as the breakthrough of the year 2013 by *Science* (1).

So far, the vast majority of efforts aimed at utilizing the immune system to reject cancer have focused on components of adaptive immunity, including monoclonal antibodies and αβT cells. The human immune system can theoretically generate up to 10^11^ unique antibodies and some 10^15^ unique αβT cell receptors (αβTCRs) (2), and controlling this vast diversity in antigen specificity for targeted immune interventions has been a major challenge for clinical implementation. Although immunoglobulins are still used in clinical practice for untargeted protection against viral infections, such as in patients with general B-cell deficiencies, the real breakthrough in clinical immunotherapy came with mastering the genetic profile of defined monoclonal antibodies. Among the first therapeutic antibodies to directly target cancer were anti-CD20 (Rituxan or Rituximab) and anti-Her2 (Herceptin or Trastuzumab) antibodies to treat B cell leukemias and breast cancer, respectively. Treatment with these antibodies, recognizing one particular antigen with a defined affinity, has underscored the therapeutic potential of truly antigen-targeted immunotherapy, as impressive clinical benefit has been reported across studies covering the last decade (3, 4). The clinical success of these pioneering agents has in recent years led to the development and regulatory approval of additional antibodies to target various cancers (5), propelling antigen-specific antibody-based immunotherapy into mainstream cancer treatment. Similar to the evolution of clinical antibody treatment, first evidence for the anti-tumor potential of adoptively transferred αβT cells originated from the transfer of a very diverse immune population, the so called donor lymphocyte infusions, in the early 1990s, when allogeneic donor αβT cells that were infused in patients after allogeneic stem cell transplantation demonstrated potent anti-leukemia responses (6). By now, these data have been complemented by remarkable clinical results obtained with strategies that aim to mobilize the tumor-reactivity of autologous T cells in cancer patients, either by the adoptive transfer of *ex vivo* expanded tumor-infiltrating lymphocytes (TILs) (7, 8) or the infusion of monoclonal antibodies that stimulate T cell activity, such as the recently approved anti-CTLA4 antibody Ipilimumab (9, 10). Additionally, the genetic engineering of T cells with tumor-reactive αβTCRs (11, 12) or antibody-based chimeric antigen receptors (CARs) (13) has gained increasing interest in recent years, and the first clinical trials using adoptive transfer of such gene-modified T cells have demonstrated potent and lasting anti-tumor responses in selected patients (14–18).

Importantly, understanding the diversity of adaptive immune repertoires and utilizing very defined specificities for therapeutic interventions has so far been not only the success but also the downside of such therapies, resulting in highly personalized cancer care that depends on antibody-based strategies (including CAR-engineered T cells) with limited numbers of targetable tumor antigens and αβT cell products that are only clinically applicable to HLA-matched patient populations. Moreover, clinical anti-tumor efficacy of αβT cell-based approaches is so far mainly restricted to particularly immunogenic tumor types, such as melanoma. Thus, there is a compelling need to call to arms alternative immune components for novel cancer immunotherapeutic concepts.

## γδT Cells: The Promising Outsiders

Unconventional γδT cells, a second lineage of T cells that express a unique somatically recombined γδTCR, possess unique features to confront the limitations of adaptive-based immunotherapeutic strategies. γδT cells are rapidly activated upon encounter of pathogen-derived antigens or self molecules that are upregulated on infected or stressed cells, resembling the activation of innate immune cells that sense molecular stress signatures (19, 20). Importantly, γδT cells are set apart from conventional αβT cells by the fact that activation of γδT cells does not depend on antigen presentation in the context of classical MHC molecules. A preferential usage of distinct TCR γ and δ chains, which together have the potential to form a tremendous repertoire of ~10^20^ uniquely recombined γδTCRs (2), has formed the basis for the identification of two major γδT cell subsets. γδT cells that carry Vγ9Vδ2^+^ TCRs are primarily found in peripheral blood, where they constitute a minor fraction of total T cells and respond to non-peptidic intermediates of the mevalonate pathway called phosphoantigens. Other γδT cells express mainly Vδ1^+^ or Vδ3^+^ chains paired with diverse γ chains (also called Vδ2^neg^ γδT cells) and are highly enriched at mucosal sites and epithelial tissues. The effector mechanisms of γδT cells are highly similar to those of αβT cells and involve the secretion of high levels of cytokines and lysis of target cells by the release of granzymes and perforin and the engagement of FAS and TRAIL death receptors. Thus, by combining the potent effector functions of adaptive αβT cells with recognition modes that target unique classes of antigens in an innate-like manner, γδT cells are regarded as valuable sentinels that bridge innate and adaptive immunity.

Underlying the interest in γδT cells for use in cancer immunotherapy is a long-standing body of evidence indicating that γδT cells play important roles in tumor immunosurveillance. Human γδT cells display potent *in vitro* cytotoxicity toward a surprisingly large array of tumors, including cells derived from both solid and hematological origin (20–22). Importantly, γδT cells are also capable of targeting chemotherapy-resistant leukemic cells (23) and to kill leukemic and colon cancer stem cells (24) and Sebestyen and Kuball, unpublished observation). *In vivo* evidence for the non-redundant relevance of γδT cells in tumor immune surveillance stems from studies showing that γδT cell-deficient mice are more susceptible for developing cancer (25–27). Moreover, tumor-infiltrating γδT cells (γδTIL) have been observed in cancer patients with various cancers, and isolated γδTILs were shown to efficiently kill autologous tumors *ex vivo*, while leaving healthy cells unharmed (28–32). Important roles for γδT cells in tumor host defense are furthermore suggested by clinical data showing that high numbers of γδTILs in tumors of melanoma patients and elevated levels of circulating γδT cells in leukemia patients correlate with increased cancer-free survival (33, 34). Taken together, these studies have established a wealth of evidence for the broad tumor-targeting capabilities of γδT cells and have sparked great interest in their application in cancer immunotherapy.

## Clinical Success of γδT Cells: Stuck in Diversity?

Given the broad recognition of unique classes of tumor antigens by γδT cells combined with their potent killing capacity, it is no surprise that γδT cells have been the focus of attempts to design novel cancer immunotherapeutic strategies. Of the two major γδT cell subsets, clinical trials conducted so far have exclusively focused on the stimulation of autologous Vγ9Vδ2^+^ γδT cells that were either activated *in vivo* using so-called aminobisphosphonate compounds that specifically activate Vγ9Vδ2^+^ γδT cells, or expanded *ex vivo* and reinfused into patients. Protocols for the *in vivo* mobilization of Vγ9Vδ2^+^ T cells generally involved repeated cycles of intravenous injection of synthetic phosphoantigen (35) or aminobisphosphonates such as pamidronate (36) or zoledronate (37–40), in combination with multiple IL2 injections per cycle. In trials that explored the adoptive transfer autologous Vγ9Vδ2^+^ T cells, patient PBMCs were cultured *ex vivo* for 2 weeks in the presence of aminobisphosphonates (41–43) or synthetic phosphoantigen (44, 45) in combination with IL2. Even though these conditions promoted the expansion of Vγ9Vδ2^+^ T cells, *ex vivo* expanded cell products contained rather low (on average 50–60%) and highly variable percentages of Vγ9Vδ2^+^ T cells, and no additional purification of Vγ9Vδ2^+^ T cells was performed prior to reinfusion into patients. Patients received repeated infusions of expanded cells, in some trials in combination with IL2. Treatment using γδT cells was generally found to be safe using both *in vivo* and *ex vivo* stimulation protocols, but clinical responses varied widely across trials and were generally limited, even in patients with cancers generally sensitive to immune responses such as renal cell carcinoma [reviewed in Ref. (46–48)]. Important limitations included (a) the need for a preselection of patients due to a wide variability in *in vitro* cytotoxicity of patient γδT cells against autologous tumor tissue (36, 41, 44), and (b) limited *in vivo* or *ex vivo* expansion potential of patient γδT cells (40, 41, 44, 45, 49). Moreover, anti-tumor efficacy of γδT cells showed only marginal improvement over standard treatment options (46). Thus, despite the fact that these trials have established the anti-tumor potential of γδT cells in cancer immunotherapy, current therapeutic strategies using these cells clearly suffer from major shortcomings that have so far prevented γδT cells to live up to their clinical promise.

## A Remarkable Diversity Hampers Application of γδT Cells in Cancer Immunotherapy

**Figure 1:**
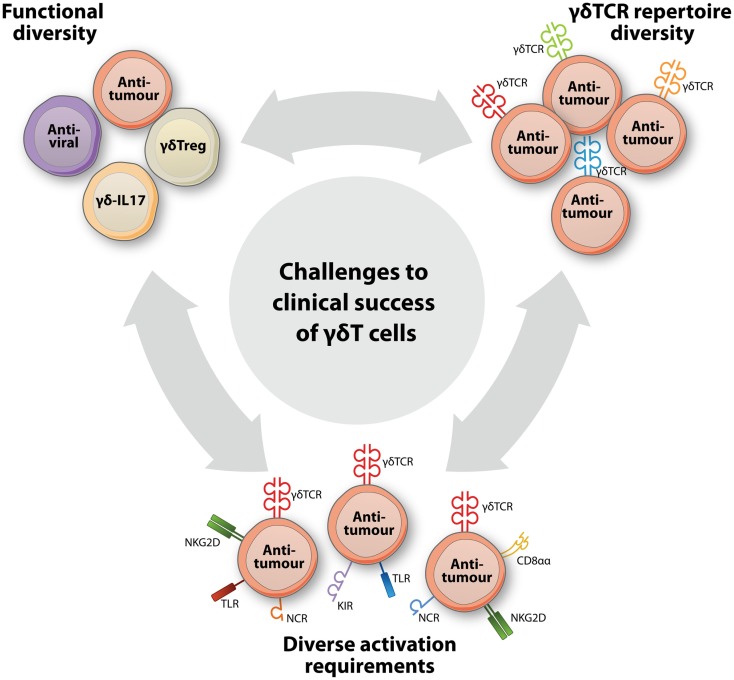
**A broad functional and clonal diversity challenges the clinical success of γδT cells in cancer immunotherapy**. New insights into γδT cell biology have pointed to at least three levels of diversity that each have a major impact on the design of successful γδT cell-based interventions to treat cancer. A striking functional diversity has come to light by the identification of new γδT cell subsets, such as regulatory (γδTreg) and IL17-producing (γδ-IL17) γδT cells, that now complement the well-established subsets with antiviral or anti-tumor functions. Within γδT cell populations that perform identical functions, another level of diversity is created by the extraordinarily diverse γδTCR repertoire that results in considerable variation in functional avidities of individual γδT cells. Additional diversity within and across γδT cell populations is represented by variable expression patterns of and complex activation requirements for additional immune receptors, including TLRs, CD8αα, and NK cell receptors such NKG2D, the natural cytotoxicity receptors (NCR) NKp30, NKp44, and NKp46, and activating and inhibitory killer cell immunoglobulin-like receptors (KIRs).

Recent years have seen important progress in the understanding of γδT cell immunobiology and have uncovered a striking diversity in γδT cell functions and subsets. These new insights have important implications for the use of γδT cells in the treatment of cancer. To date, however, a profound appreciation of this γδT cell diversity has lacked from γδT cell-based clinical concepts and this is likely to contribute to the limited clinical results observed so far. At least three levels of γδT cell heterogeneity can be distinguished (Figure 1), including (a) a multitude of immune functions mediated by γδT cells, (b) a diverse γδTCR repertoire that, also for similar antigen-specificities, mediates different affinities, and (c) the complex and diverse molecular needs for target recognition within the same and across different γδT cell populations. A thorough consideration of these features will be of central importance to improving the clinical efficacy of γδT cells in treating cancer.

### γδT cell functions: The more the better?

γδT cells have, as discussed above, been attributed important and valuable functions in tumor immunosurveillance, but reactivity toward tumors is far from the only part that γδT cells play in immunity. By now, it is evident that γδT cells perform a plethora of functions that underline their involvement in diverse pathophysiological conditions other than cancer, including host defense against infectious pathogens such as bacteria, viruses, and parasites, the modulation of the activity of other immune cells, and promoting tissue regenerating after injury (20, 50).

Rapid expansions of γδT cells are observed in human beings infected with a variety of viruses or bacteria and γδT cells possess a potent capacity to directly kill infected cells (51). Moreover, a proportion of γδT cells contribute to pathogen clearance by the secretion of anti-microbial peptides such as granulysin and cathelicidin (52–54). Intriguingly, the recognition of pathogens may have important implications for γδT cell-mediated cytotoxicity against cancers, as subsets of γδT cells that respond to cytomegalovirus (CMV) infection have been reported to cross-recognize solid (55) as well as hematological (56) tumor cells *in vitro*. A role for virus-induced γδT cells in the protection from cancer *in vivo* is supported by observations that CMV infection in kidney transplant recipients was observed to associate with increased levels of γδT cells and concomitantly a reduced risk of developing cancer (57). Also in leukemia patients treated with hematopoietic stem cell transplantation, CMV infection associates with lower incidence of leukemic relapse after transplantation (58, 59) and work from our laboratory has demonstrated that tumor surveillance by CMV-induced γδT cells is likely to play a major role in this (56), emphasizing the clinical value of such dual-reactive γδT cells in immunotherapy.

In addition to their strong reactivity to a wide variety of tumors and pathogens, a valuable feature of γδT cells is their capability to broaden immune responses by recruiting and activating additional immune cell populations. For example, activated γδT cells have the potential to orchestrate adaptive αβT cell responses, both directly by functioning as antigen-presenting cells (60–62) as well as indirectly via the interaction with dendritic cells (56, 63, 64). In addition, γδT cells have been reported to secrete cytokines to provide B cell help in the production of antibodies (65, 66), to prime NK cells to kill tumor cells (67), to rapidly recruit neutrophils via the secretion of IL-17 (68, 69), and to synergize with monocytes to mount anti-microbial αβT cell responses (70). However, in addition to the immunostimulatory roles of γδT cells, their modulatory function may be of regulatory nature as well, suggesting complex implications of γδT cells in mediating broader immune responses. For example, depending on antigenic exposure, γδT cells may suppress rather than promote antibody production by B cells (71, 72). Similarly, γδT cells can strongly inhibit the proliferation of activated αβT cells (73, 74), and a suboptimal maturation of DCs by γδT cells (56) may induce tolerogenic rather than cytotoxic αβT cell responses. Importantly, human and mouse IL17-producing γδT cells have recently been demonstrated to facilitate tumor growth by recruiting myeloid-derived suppressor cells to tumor sites (75, 76). With the recent identification of bona fide Foxp3-expressing regulatory γδT cell subsets (77), it is thus becoming clear that, depending on their local or temporal cytokine milieu, activated γδT cells may suppress instead of activate local immune responses (78). Indeed, even though the presence of γδT cells may correlate with increased survival of cancer patients in some studies (see above), their infiltration into tumor sites may also associate with worse clinical outcome of patients due to a immunosuppressive phenotype of local γδT cells (79–81).

### A very diverse γδTCR repertoire produces receptors with variable anti-tumor affinities

Like αβTCRs and B cell receptors, γδTCRs are generated during T cell maturation through the somatic recombination of germline-encoded variable (V), diversity (D), and joining (J) gene segments. Despite the fact that the number of germline Vγ and Vδ genes is far more limited than the repertoire of Vα and Vβ genes, more extensive junctional diversification processes during TCR γ and δ chain rearrangement leads to a potential γδTCR repertoire that is roughly 10^5^-fold larger than that of αβTCRs (2). Despite this extensive γδTCR repertoire, the diversity of antigens that are recognized by γδTCRs appears to be surprisingly limited. The vast majority of Vγ9Vδ2^+^ TCRs on circulating γδT cells are restricted to sensing elevated levels of phosphoantigens (22, 82), a process that has recently been demonstrated to involve the butyrophilin family member BTN3A1 (83, 84). Similarly, all antigens of Vδ2^neg^ γδTCRs identified so far, including MICA/B (85), CD1 (86, 87), and EPCR (88), belong to the family of non-classical MHC homologs, although additional antigens are likely to still be identified and may include MHC-unrelated molecules.

An important question is why this rather narrow antigen restriction of γδT cells is confronted with such a broad γδTCR diversity, instead of a rather oligoclonal or invariant repertoire as expressed by for example NKT cells (89). One possible explanation may be that the extensive γδTCR repertoire of γδT cells allows an important fine-tuning of γδTCR-mediated target cell recognition. Indeed, we have shown recently that phosphoantigen-responsive Vγ9Vδ2^+^ γδT cell clones differed widely in their functional avidity toward tumor cells (90). γδTCR transfer and mutation experiments showed that this variability in the ability to respond to tumor cells was mediated primarily through diverse sequence compositions that dictate the affinities of individual clone-derived Vγ9Vδ2^+^ TCRs. A similar γδTCR-mediated heterogeneity in anti-tumor specificity can be observed in the Vδ2^neg^ subset of γδT cells, as we recently demonstrated that individual Vδ1^+^ γδT cell clones display γδTCR-mediated reactivity against diverse arrays of tumor cells (56). Moreover, γδTCRs of other Vδ1^+^ clones were not involved in tumor recognition but mediated interactions with dendritic cells, demonstrating that a diverse γδTCR repertoire can mediate not only a fine-tuning of anti-tumor avidity but also different functions. Accordingly, diverse γδT cell functions that segregate with γδTCR composition have been observed for the human Vγ9Vδ2^+^ and Vδ2^neg^ subsets, as Vγ9Vδ2 γδT cells have been generally ascribed potent cytotoxic effector functions, while Vδ2^neg^ γδT cells rather have immunomodulatory roles (91, 92). However, these observations are contrasted by reports showing a superior tumor-homing and -killing capacity of Vδ2^neg^ γδTILs over Vγ9Vδ2 γδTILs in some cancers (30, 93), further underlining the heterogeneous and context-dependent nature of both γδT cell subsets.

### γδT cell activation: A complex interplay between receptors

Alongside the γδTCR, γδT cells can be activated through a variety of activating and inhibitory NK receptors (48, 94) and toll-like receptors (TLR) (95), emphasizing the innate-like nature of these unconventional T cells. Depending on the pathophysiological context, these receptors can provide costimulation to γδTCR-mediated activation signals or can activate γδT cells independent of γδTCR triggering, adding yet another level of heterogeneity and complexity to γδT cell biology. The best-studied receptor with dualistic roles in γδT cell activation is NKG2D, a natural cytotoxicity receptor (NCR) that is expressed on NK cells, most γδT cells and CD8^+^ αβT cells. NKG2D recognizes the non-classical MHC homologs MICA/B and ULBPs, the expression of which is upregulated on many different tumors (96, 97). On Vγ9Vδ2^+^ γδT cells, NKG2D can amplify γδTCR-mediated effector functions in response to MICA/B-positive target cells (98, 99). In other cases, however, sole signaling through NKG2D has been proposed to be sufficient for activating γδT cells, without requiring γδTCR engagement (100, 101). However, as most of these studies have used TCR blocking antibodies and not receptor gene-transfer experiments, the impact of TCR affinity and signaling in NKG2D-triggered γδT cell activation might have been underestimated (Gründer and Kuball, unpublished observation). Factors that determine the directly stimulatory versus costimulatory function of NKG2D are not known, but may involve signaling by polymorphic receptors such as inhibitory NK receptors (100). Apart from serving as ligand for NKG2D, MICA/B is also recognized by selected Vδ1^+^ γδTCRs (85). In fact, overlapping binding epitopes for NKG2D and γδTCRs on MICA/B result in competitive binding of both receptors for MIC ligands, suggestive of complex, temporally regulated interactions of both receptors for MIC ligands (102). Similarly, engagement of the NCRs NKp30, NKp44, and NKp46 on γδT cells can be sufficient for eliciting anti-tumor cytotoxicity, but interestingly only after expression of these receptors on γδT cells has been induced via triggering of the γδTCR (103). Differential involvement of the γδTCR and additional receptors has also been reported in pathophysiological processes other than cancer, as work by us and others has demonstrated that reactivity of γδT cells against CMV-infected cells may involve γδTCR-dependent (55, 104) and -independent (56) pathways, suggesting multimodal pathogen-sensing mechanisms that may involve NK receptors (48).

Recently, we have found additional evidence for a complex interplay between receptors in the response of γδT cells against tumor cells by demonstrating that CD8αα, that serves as coreceptor for selected γδTCRs as reported by us recently (56), mediates γδTCR costimulation in a manner that depends on the particular tumor cell target (Scheper and Kuball, unpublished observation). Expression of CD8αα on T cells engineered to express a tumor-reactive γδTCR was a prerequisite for recognition of all tested tumor cell lines, but coexpression of signaling-deficient CD8α variants or mutants with single residue substitutions in the extracellular domain of CD8α alongside the γδTCR differentially impacted T cell reactivity toward the different tumor targets. Even though CD8αα+ γδT cells were first identified over 20 years ago, when CD8αα was found to be commonly expressed on Vδ1^+^ γδT cells in the intestine but not circulating Vγ9Vδ2^+^ T cells (105, 106), the functional implications of CD8αα expression on γδT cells have remained rather controversial. A number of studies have reported regulatory functions for CD8αα^+^ γδT cells, being capable of for example inhibiting inflammatory responses in celiac disease (107) but also to suppress αβT cell-mediated responses against tumor cells (80). On the other hand, and in line with our data (56), stimulated CD8αα^+^ γδT cells have been reported to be as capable as CD8αα^−^γδT cells of secreting high levels of Th1 cytokines such as IFNγ (108). Moreover, cytokines produced by CD8αα^+^ but not CD8αα^−^ γδT cells have been implicated in the controlling of R5-tropic HIV replication and persistence (109). Thus, CD8αα^+^ γδT cells appear to perform diverse functions depending on the context in which they are activated.

Taken together, the emerging insights into the molecular requirements for γδT cell activation and the interplay between different receptors in this process have substantially furthered our understanding of the response of γδT cells against cancer cells, but also unveil substantial challenges to the design of uniform γδT cell-based strategies for cancer immunotherapy.

## Successful Translation Using γδT Cells: Picking the Right Ones

Beyond doubt, the implications of the functional and clonal heterogeneity of γδT cells for their application in the treatment of cancer are substantial, and a failure to fully recognize this diversity in clinical concepts and trial designs is likely the most important contributing factor in the limited clinical results observed with γδT cells to date. Current clinical protocols based on the broad activation of unselected γδT cells are likely to induce γδT cell populations with diverse specificities, avidities, and functions, including regulatory. Consequently, high-avidity γδT cells with strong tumor-reactivity and a desired functional profile may represent only a relatively minor population of such cell products. In addition, stimulation of γδT cells using agents that primarily depend on strong γδTCR-mediated activation, such as the use of aminobisphosphonate and phoshoantigen compounds to expand Vγ9Vδ2^+^ γδT cells in trials pursued to date, most likely selects for γδT cells with low affinity Vγ9Vδ2^+^ γδTCRs and thus, low activity on primary tumor cells. Moreover, γδTCR-based activation strategies do not necessarily mobilize γδT cells that express a repertoire of NK receptors and TLRs required to potently respond to the multimolecular stress signature of tumor cells. Thus, the selection of optimally tumor-reactive γδT cell populations will likely be a critical parameter in the design of improved cancer immunotherapeutic concepts. In principal, this would favor strategies aimed at *ex vivo* rather than *in vivo* expansion of γδT cells, since the first allows a careful monitoring and culture-dependent skewing of γδT cell phenotype and functionality that is far more challenging to accomplish using *in vivo* stimulation protocols. With the clinical data available so far, it is difficult to corroborate this by comparing clinical responses observed in both types of trials, as studies using adoptive transfer of *ex vivo* generated γδT cells have so far relied on similar stimulation protocols (aminobisphosphonate or phosphoantigen in combination with IL-2) and the potential for extended *in vitro* manipulation for enhanced anti-tumor efficacy has not yet been investigated (41–45, 49, 110). Importantly, *ex vivo* manipulation of patient γδT cells could also include a valuable enrichment of tumor-specific γδT cells with high functional avidity, for instance using selection techniques based on the upregulation of activation markers or the production of cytokines such as IFNγ by γδT cells after *in vitro* coculture with autologous tumor cells. Nevertheless, γδTCR repertoires vary widely among individuals (111, 112), and generating sufficient numbers of γδT cells that recognize tumors with high avidity may therefore be challenging in certain patients. Similarly, NK receptor and TLR repertoires as well as CD8α expression levels differ considerably between γδT cell subsets (56, 103, 105, 113) and between individuals (95, 114, 115), putting additional constraints on the generation of γδT cell products potently capable of rejecting cancer.

To overcome the limitations of patient γδT cell repertoires, γδTCRs with broad tumor-specificity could be identified *in vitro* and genetically introduced into patient-derived immune cells. Recent work by our group has demonstrated that gene-transfer of tumor-specific Vγ9Vδ2^+^ and Vδ1^+^ γδTCRs can be used to efficiently reprogram conventional αβT cells to recognize a wide variety of tumor cells (56, 90, 97). By exploiting the abundance and superior proliferation potential of αβT cells, large numbers of autologous γδTCR-engineered T cells with defined tumor-specificity can be generated *ex vivo* and subsequently reinfused into cancer patients. In contrast to αβTCR gene-transfer strategies, introduced TCR γ and δ chains do not dimerize with endogenous αβTCR chains (97) and therefore do not lead to the formation of unwanted TCRs with unpredictable, and potentially dangerous, specificities. Moreover, since antigen recognition by γδTCRs does not depend on classical MHC molecules, well-characterized γδTCRs that mediate superior anti-tumor functional avidities can be applied to a broad patient population without the requirement for HLA matching. Additionally, transgenic expression of γδTCRs downregulates surface expression of endogenous αβTCR chains (56, 90, 97), enabling the use of engineered cell product even in an allogeneic “off-the-shelf” fashion. The *ex vivo* generation of γδTCR-engineered T cells furthermore allows additional manipulation of cell products, such as the selection of T cells with highest γδTCR expression levels or T cells which express beneficial TLRs or NK receptors. Importantly, such strategies can take advantage of the valuable lessons that have been learned from efforts to apply conventional αβT cells and their receptors in cancer immunotherapy, such as evidence for the effect of the differentiation status on *in vivo* persistence and function of clinical T cells (116). Our group has initiated the first clinical trial using γδTCR-gene-modified T cells to treat cancer patients (scheduled to start in 2015). Donor T cells engineered with a well-characterized tumor-reactive Vγ9Vδ2^+^ γδTCR (90) will be administered to leukemia patients after allogeneic stem cell transplantation as part of an engineered donor lymphocyte infusion. *Ex vivo* manipulations of gene-modified T cell products will include the depletion of cells that express only low levels of the clinical γδTCR and adapted culturing conditions to prevent terminal differentiation of engineered T cells before infusion into patients.

## Closing Remarks

Even though γδT cells have traditionally been regarded as a homogeneous immune population, important advances in the understanding of γδT cell immunobiology have revealed a striking diversity in functionality and molecular activation modes. These new insights are generally met with great enthusiasm as they give acclaim to γδT cells for their non-redundant involvement in so many pathophysiological and homeostatic processes. However, this pleiotropy of γδT cells is likely an important factor that stifles the clinical success of their application to treat cancer. As for adaptive immune interventions, it may be absolutely mandatory to carefully consider the plethora of γδT cell functions, the diversity in γδTCR specificities and affinities as well as the complex requirements for proper γδT cell activation. At the end, such broadly tumor-reactive γδT cells might be highly effective only under very defined molecular and pathophysiological conditions and therefore less broadly applicable as initially thought, though a valuable addition to current therapeutic options. This new concept represents a major challenge in the design of next generation γδT cell-based immunotherapies, and clinical trials that incorporate these exciting insights will need to be pursued to confirm the clinical potential of γδT cells in the treatment of cancer.

## Author Contributions

Wouter Scheper, Zsolt Sebestyen, and Jürgen Kuball wrote the manuscript; all authors agreed on the final manuscript.

## Conflict of Interest Statement

The scheduled clinical trial using γδTCR-gene engineered T cells is supported by Miltenyi Biotec.
